# Comparison of Huntington’s disease phenotype progression in male and female heterozygous FDNQ175 mice

**DOI:** 10.1186/s13041-023-01054-6

**Published:** 2023-09-19

**Authors:** Si Han Li, Tash-Lynn L. Colson, Jingwei Chen, Khaled S. Abd-Elrahman, Stephen S. G. Ferguson

**Affiliations:** 1 University of Ottawa Brain and Mind Research Institute, 451 Smyth Road, Ottawa, ON K1H 8M5 Canada; 2 Department of Cellular and Molecular Medicine, Faculty of Medicine, University of Ottawa, 451 Smyth Road, Ottawa, ON K1H 8M5 Canada; 3 Department of Neuroscience, Faculty of Health Sciences, Carleton University, 1125 Colonel By Dr., Ottawa, ON K1S 5B6 Canada; 4 Department of Anesthesiology, Pharmacology and Therapeutics, and Djavad Mowafaghian Centre for Brain Health, University of British Columbia, Vancouver, BC V6T 1Z3 Canada; 5 Department of Pharmacology and Therapeutics, College of Medicine and Health Science, Khalifa University, Abu Dhabi, United Arab Emirates; 6 Department of Pharmacology and Toxicology, Faculty of Pharmacy, Alexandria University, Alexandria, 21521 Egypt

**Keywords:** Neurodegenerative diseases, Huntingtin, Sex differences, Mouse model, Motor function

## Abstract

Huntington’s Disease (HD) is an inherited autosomal dominant neurodegenerative disorder that leads to progressive motor and cognitive impairment due to the expansion of a polyglutamine (CAG) repeat in the N-terminal region of the huntingtin (Htt) protein. The creation of HD mouse models represents a critical step in the research for HD treatment. Among the currently available HD mouse models, the zQ175 knock-in mouse line is the first to display robust disease phenotype on a heterozygous background. The newer FDNQ175 mouse model is derived from the zQ175 mouse line and presents a more aggressive phenotype. Moreover, increasing evidence has implicated sex as a contributing factor in the progression of HD symptoms. Here, we compared the progression of HD phenotypes in male and female heterozygous FDNQ175 mice. We found that both male and female heterozygous mice showed deficits in forelimb grip strength and cognition as early as 6 months of age. However, female FDNQ175 mice were less vulnerable to HD-associated decline in limb coordination and movement. Neither male nor female FDNQ175 mice exhibited reduced locomotor activity in the open field or exhibit consistent differences in anxiety at 6–12 months of age. Both male and female FDNQ175 mice exhibited increased numbers of huntingtin aggregates with age and 8-month-old female FDNQ175 mice had significantly more aggregates than their male counterparts. Taken together, our results provide further evidence that sex can influence the progression of HD phenotype in preclinical animal models and must be taken into consideration for future HD research.

## Introduction

Huntington’s disease (HD) is an inherited autosomal dominant neurodegenerative disorder characterized by the early loss of medium spiny neurons in the striatum [1]. HD symptoms typically manifests between the age of 30–50 and includes choreatic movements, dementia and behavioural difficulties [2]. HD is caused by the expansion of a polyglutamine (CAG) repeat in the N-terminal region of the huntingtin (Htt) protein [3]. Mutant Htt with this expanded polyglutamine tract has been shown to be targeted for proteolysis and the cleavage of Htt at the N-terminus results in the formation of cytoplasmic and intranuclear aggregates that strongly correlate with HD symptoms and severity [4]. Indeed, longer polyglutamine repeats are associated with earlier disease onset and more severe symptoms [5, 6]. Despite this well-characterized etiology, the underlying molecular mechanism(s) responsible for HD pathogenesis remains poorly understood and there is no disease modifying treatment currently available.

One important advance in HD research is the creation of genetic mouse models that replicate the clinical, pathological and biochemical changes observed in HD patients. These different mouse models present varying degrees of phenotype severity, pathology and onset. Based on the way in which mutant huntingtin is incorporated into the mouse genome, genetic models of HD can be broadly separated into three categories: (i) transgenic mice that express toxic N-terminal fragments of human mutant Htt (mHtt) in addition to mouse wild-type Htt, (ii) transgenic mice that express full length human mHtt alongside mouse wild-type Htt, and (iii) knock-in mice with expanded CAG repeats in their endogenous Htt gene [7]. Among the three categories, knock-in mouse lines represent the most genetically accurate models of HD, as they express the disease-causing mutant protein in the correct genomic context. zQ175 mouse is a knock-in mouse model of HD that expresses a chimeric Htt protein containing exon 1 of mutated human Htt with roughly 188 CAG repeats. Importantly, zQ175 mice show robust HD phenotype on a heterozygous back ground, which closely recapitulates the autosomal dominant inheritance pattern of HD in humans [8]. More recently, the FDNQ175 mouse model was generated to increase phenotype severity and reduce testing time. These improvements are achieved by changing the background strain from C57BL/6 to FVB/N and removing the floxed neomycin resistance gene (Neo) upstream of the Htt locus, which increased the animal’s susceptibility to neurodegeneration and elevates mutant Htt protein levels in the brain [9].

The initial assessment of FDNQ175 mice suggests that it is a more suitable model for HD preclinical testing than zQ175 mice. However, the original study did not distinguish between the severity and progression of the phenotype in males and females. There is growing evidence that sex may influence HD phenotype and neuropathology in HD rodent models and patients [10, 11]. Therefore, we examined and compared the progression of HD phenotypes in male versus female FDNQ175 mice. We found that female FDNQ175 mice appeared less vulnerable to HD-associated decline in motor function than male mice. Interestingly, female wild-type mice developed deficits in the novel object recognition test at 8 months of age, whereas male wild-type mice only showed deficits in the same test at 12 months of age. Moreover, female FDNQ175 mice appears to have more insoluble aggregates than male mice at younger ages, but they eventually reach a similar level by 12 months of age. Overall, these studies highlight significant differences in disease progression between male and female FDNQ175 mice.

## Materials and methods

### Animals

All animal experimental protocols were approved by the University of Ottawa Institutional Animal Care Committee and were in accordance with the Canadian Council of Animal Care guidelines. Animals were group caged and housed under a constant 12-hour light/dark cycle. Food and water were given *ad libitum*. Wild-type FVB/N mice were from Charles River Laboratory (#207) and FDNQ175 mice were gifts from Michael Hayden’s laboratory (University of British Columbia). Mice were bred to establish littermate-controlled male and female wild-type and heterozygous FDNQ175 mice. Groups (n ≥ 14) of male and female wild-type and heterozygous FDNQ175 mice were aged and tested in a series of behavioural experiments at 6, 8, 10 and 12 months of age. After 12 months, mice were sacrificed by live cervical dislocation and the brains were collected for biochemistry and immunohistochemistry. A second group of male and female wild-type and heterozygous FDNQ175 mice were raised and sacrificed by live cervical dislocation at 8 months of age and the brains were also collected for immunohistochemistry.

### Behavioural analysis

All animals were habituated in the testing room for a minimum of 30 min before testing. All behavioural tests were performed blindly and during the animal’s dark cycle. Male and female mice were tested separately to prevent change in behaviour. All testing was performed in the University of Ottawa Behavior and Physiology Core Facility.

### Forelimb grip strength test

The grip strength of each mouse was measured using the Chatillon DEF II Grip Strength Meter (Columbus Instruments, Columbus, Ohio). Mice were held over the grid of the instrument by their tails and allowed to firmly grip the bar. The mice were then pulled horizontally away from the bar using constant force and at a speed of ~ 2.5 cm/s until they released the bar. Each mouse was tested 8 times with a break of 5 s in between each trial and the values of maximal peak force were recorded.

### Open field test

Mice were individually placed in the bottom-left corner of an opaque and illuminated (~ 300 lux) open field arena (45 cm × 45 cm × 45 cm) and allowed to explore for 10 min. Activity of the mice were recorded by an overhead camera connected to a computer outside the room. Total distance travelled, velocity and time spent in the center versus four corners were calculated using the Noduls Ethovision 17 software.

### Rotarod test

Mice were introduced to the rotarod apparatus (IITC, Woodlands Hills, CA, USA) on day one by placing them on the stationary rotarod for 3 min. Four trials were then performed daily for two consecutive days using an accelerating protocol (from 5 to 45 RPM in 300 s) with 10 min of rest between each trial. Any mice remaining on the rotarod after 300 s were scored as 300 s. If mice fell in the first 10 s of a trial, the trial was repeated from the start, for up to three times. Data obtained from the four trials of the second day were used for analysis.

### **Horizontal ladder test**

The mice were first trained (1 trial) to traverse a horizontal ladder with a total of 121 regularly (1 cm apart) and irregularly (0.5–2.5 cm apart) spaced metal rungs (0.15 cm in diameter and 2 cm from the bottom of the wall). The mice were then filmed crossing the ladder for 4–5 trials using high-definition camera. The number of successful and missed steps for each limb during the 2 best trials were quantified and interpreted as percentage error.

### Immunohistochemistry

One hemisphere of each brain sample was fixed in 4% paraformaldehyde for 48 h and then transferred to 70% ethanol for storage at 4 °C. The samples were embedded in paraffin and then coronally sectioned through the striatum at a thickness of 5 μm. Sections were then incubated with the mouse monoclonal EM48 antibody (Sigama-Aldrich, MAB5374) at 1:100 dilution for 30 min at room temperature and staining was done using Leica Bond III automatic stainer using BOND polymer Refine Detection Kit (Leica Biosystems Cat# DS9800) from Leica Biosystems. Slides were scanned using a Leica Aperio Slide scanner at 20X and the number of EM48 positive aggregates were counted in representative 300 × 300 µm^2^ areas of the striatum. Experimenters were blinded to analysis and six sections per mouse were analyzed and for each Sect. 2 ROIs in the striatum were quantified using the cell counter tool in Image J [12–15].

### Statistical analysis

Means ± SEM for each independent experiment is shown in the various figure legends. GraphPad Prism software was used to analyze the data for statistical significance. Statistical significance was determined by a series of 2 (strain) × 2 (sex) or 2 (strain) × 2 (age) analyses of variance (ANOVAs), followed by Fisher’s least significant difference comparisons for the significant main effects or interactions.

## Results

### Male and female heterozygous FDNQ175 mice develop deficits in grip strength at 6 months of age

**Fig. 1:**
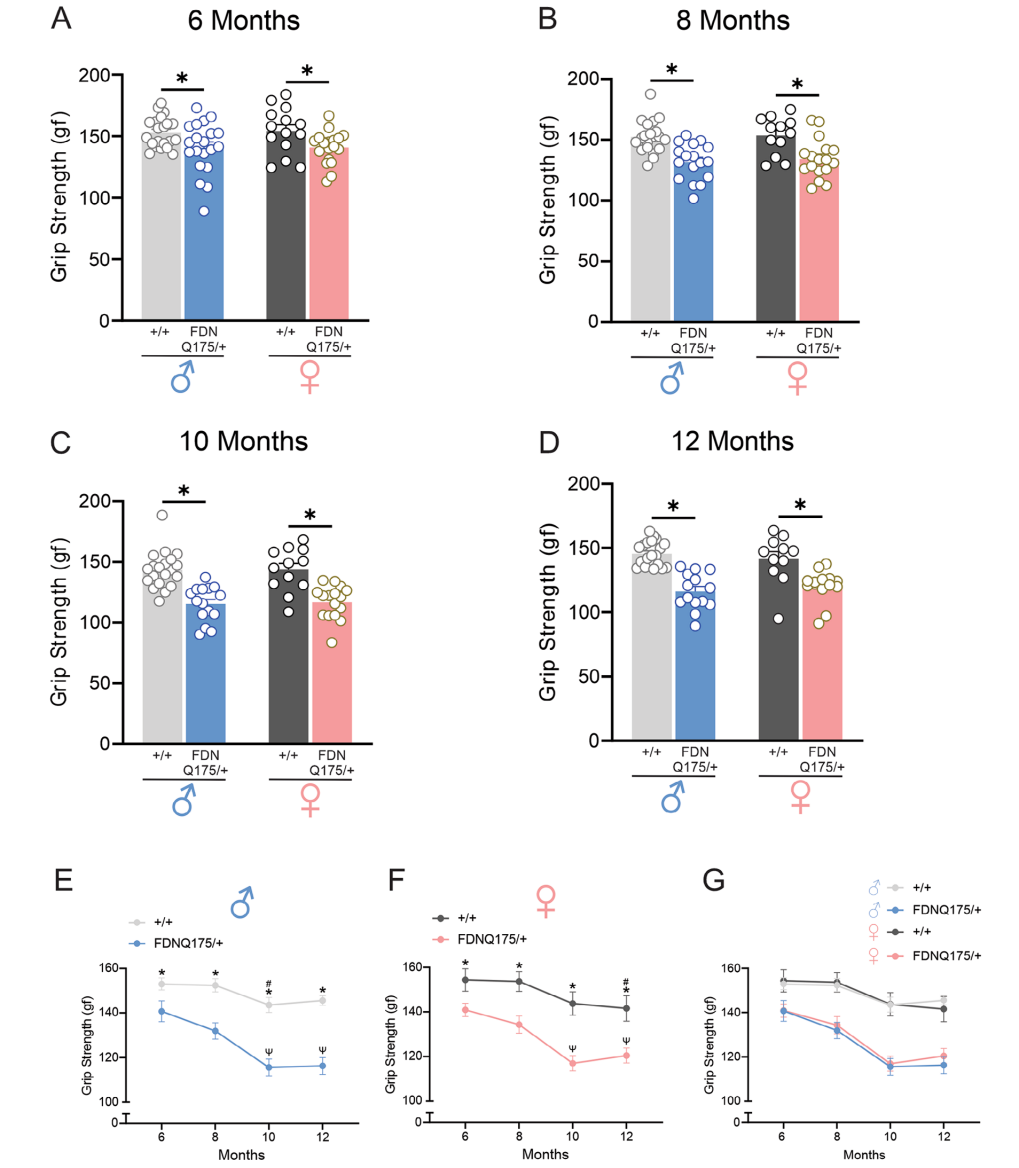
Fore limb grip strength of male and female wild-type and FDNQ175 mice from 6 to 12 months of age. Mean ± SEM grip strength [gram-force (gf)] of wild-type (+/+) and heterozygous FDNQ175 (FDNQ175/+) male and female mice at 6 months (n = 14–21 per group) **(A)**, 8 months (n = 12–20 per group) **(B)**, 10 months (n = 12–20 per group) **(C)**, and 12 months (n = 11–20 per group) **(D)** of age. **(E)** Line graph summarizing changes in the mean ± SEM grip strength [gram-force (gf)] of wild-type (+/+; n = 20–21) and heterozygous FDNQ175 (FDNQ175/+; n = 14–20) male mice from 6 to 12 months of age tested at 2 month intervals. **(F)** Line graph summarizing changes in the mean ± SEM grip strength [gram-force (gf)] of wild-type (+/+; n = 11–14) and heterozygous FDNQ175 (FDNQ175/+; n = 14–19) female mice from 6 to 12 months of age tested at 2 month intervals. **(G)** Combined line graph summarizing changes in the mean ± SEM grip strength [gram-force (gf)] of wild-type (+/+) and heterozygous FDNQ175 (FDNQ175/+) male and female mice from 6 to 12 months of age tested at 2month intervals. * indicates significant difference (p < 0.05) between age-matched and sex-matched wild-type (+/+) and heterozygous FDNQ175 (FDNQ175/+) mice. ^#^ indicates significant difference (p < 0.05) as compared with 6-month-old sex-matched wild-type (+/+) mice. ^Ψ^ indicates significant difference (p < 0.05) as compared with 6-month-old sex-matched heterozygous FDNQ175 (FDNQ175/+) mice

It was previously reported that heterozygous zQ175 mice develop deficits in motor function at approximately 8 months of age [8]. In comparison, FDNQ175 mice showed decline in motor function starting at 6 months of age [9]. In this study, we tested both male and female FDNQ175 mice to determine whether they develop HD-related motor impairments in a sex-specific manner. At 6 months of age, both male and female heterozygous FDNQ175 mice showed lower grip strength than age and sex-matched wild-type controls (Fig. 1A). Furthermore, the grip strength of heterozygous FDNQ175 mice continued to deteriorate as they aged, with additional decline occurring from 6 to 10 months (Fig. 1B-F). Interestingly, we did not observe a further drop in grip strength between the age of 10 and 12 months, suggesting that the loss of grip strength had already plateaued at the 10 months time point for FDNQ175 mice (Fig. 1B-F). We also did not observe significant differences in grip strength between wild-type male and female mice nor between heterozygous male and female FDNQ175 mice at each time point tested in this study (Fig. 1G). In conclusion, both male and female heterozygous FDNQ175 mice exhibited significant decline in their grip strength at 6 months of age. Moreover, their grip strength continued to decrease further up to 10 months of age. However, no evidence of any sex-specific differences in the development of grip strength dysfunction was observed in FDNQ175 mice.

### Male and female heterozygous FDNQ175 mice develop deficits in limb coordination and placements in a sex-dependent manner

**Fig. 2:**
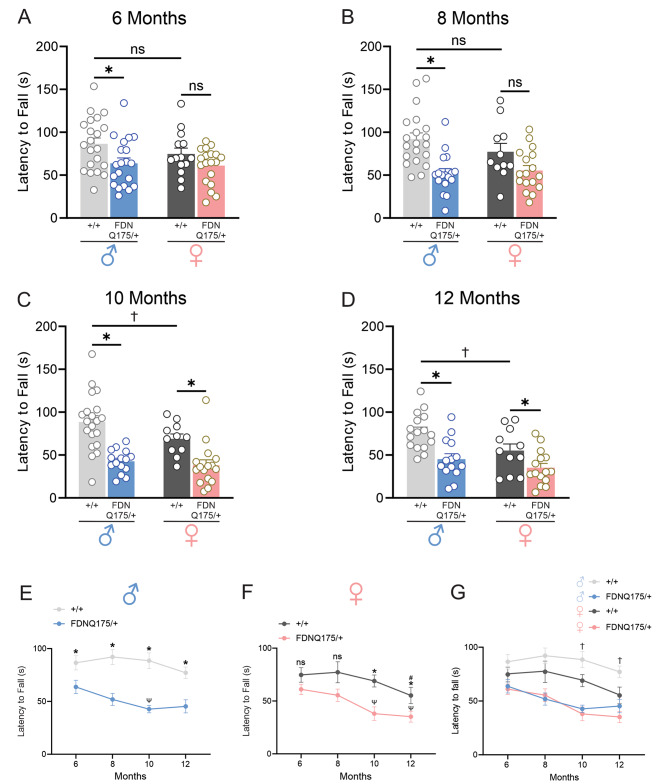
Rotarod performance of male and female wild-type and FDNQ175 mice from 6 to 12 months of age. Mean ± SEM latency to fall (s) from accelerating rotarod of wild-type (+/+) and heterozygous FDNQ175 (FDNQ175/+) male and female mice at 6 months (n = 14–21 per group) **(A)**, 8 months (n = 11–20 per group) **(B)**, 10 months (n = 11–20 per group) **(C)**, and 12 months (n = 11–16 per group) **(D)** of age. **(E)** Line graph summarizing changes in the mean ± SEM latency to fall (s) of wild-type (+/+; n = 16–21) and heterozygous FDNQ175 (FDNQ175/+; n = 14–20) male mice from 6 to 12 months of age tested at 2 month intervals. **(F)** Line graph summarizing changes in the mean ± SEM latency to fall (s) of wild-type (+/+; n = 11–14) and heterozygous FDNQ175 (FDNQ175/+; n = 15–19) female mice from 6 to 12 months of age at tested 2 month intervals. **(G)** Combined line graph summarizing changes in the mean ± SEM latency to fall (s) of wild-type (+/+) and heterozygous FDNQ175 (FDNQ175/+) male and female mice from 6 to 12 months of age at tested 2-month intervals. * indicates significant difference (p < 0.05) between age-matched and sex-matched wild-type (+/+) and heterozygous FDNQ175 (FDNQ175/+) mice. ^#^ indicates significant difference (p < 0.05) as compared with 6-month-old sex-matched wild-type (+/+) mice. ^Ψ^ indicates significant difference (p < 0.05) as compared with 6-month-old sex-matched heterozygous FDNQ175 (FDNQ175/+) mice. † indicates significant difference (p < 0.05) between age-matched male and female wild-type (+/+) mice

We previously detected deficits in rotarod and horizontal ladder performance in both male and female heterozygous zQ175 mice at 12 months of age [15, 16]. Another study had shown that heterozygous zQ175 mice can develop impairments in rotarod performance as early as 7 months [8]. In the current study, we examined whether similar deficits would develop at an earlier age in the heterozygous FDNQ175 mouse model. The rotarod performance of male heterozygous FDNQ175 mice was significantly worse than that of male wild-type mice at 6 and 8 months of age (Fig. 2A). In contrast, the rotarod performance of female heterozygous FDNQ175 mice remained comparable to age-matched female wild-type controls at the 6- and 8-month time points (Fig. 2A and B). Starting from 10 months of age, both male and female heterozygous FDNQ175 mice showed significant deficits in rotarod performance compared to their respective age- and sex-matched wild-type controls (Fig. 2C and D). Furthermore, the rotarod performance of male and female heterozygous FDNQ175 mice deteriorates between the age of 6 and 10 months (Fig. 2E and F). Importantly, the rotarod performance of age-matched male and female heterozygous FDNQ175 mice were similar at all 4 time points tested (Fig. 2G). Instead, female wild-type mice appeared to perform worse than age-matched male wild-type mice. Specifically, the rotarod performance of female wild-type mice also shows a downward trend after the 8-month time point and is significantly worse than that of age-matched male wild-type mice at the 10- and 12-month time points (Fig. 2C, D and G). In fact, 12-month-old wild-type female mice showed a significant decrease in their rotarod performance compared to their 6-month data (Fig. 2F). However, female wild-type mice at 12 months of age still performed significantly better than age-matched female heterozygous FDNQ175 mice (Fig. 2D and F). Because female wild-type mice showed a lower starting level of performance on the rotarod than male wild-type mice, it is difficult to conclude whether the development of deficits in the rotarod test is sex-dependent in FDNQ175 mice.

**Fig. 3:**
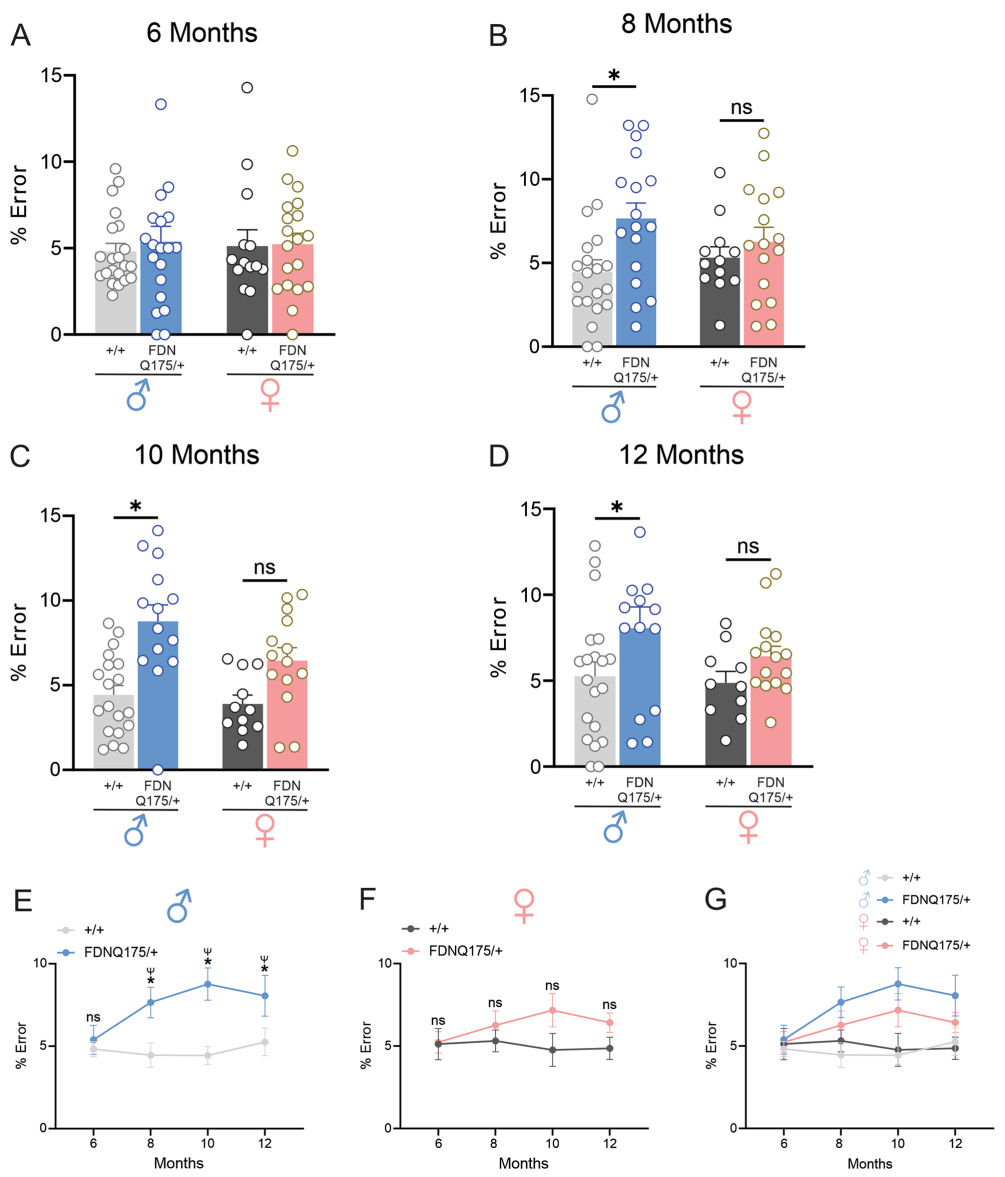
Horizontal ladder rung test performance of male and female wild-type and FDNQ175 mice from 6 to 12 months of age. Mean ± SEM percent error (% error) in limb placement while crossing the horizontal ladder of wild-type (+/+) and heterozygous FDNQ175 (FDNQ175/+) male and female mice at 6 months (n = 14–21 per group) **(A)**, 8 months (n = 12–20 per group) **(B)**, 10 months (n = 11–19 per group) **(C)**, and 12 months (n = 10–20 per group) **(D)** of age. **(E)** Line graph summarizing changes in the mean ± SEM percent error (% error) in limb placement of wild-type (+/+; n = 19–21) and heterozygous FDNQ175 (FDNQ175/+; n = 14–20) male mice from 6 to 12 months of age tested at 2 month intervals. **(F)** Line graph summarizing changes in the mean ± SEM percent error (% error) in limb placement of wild-type (+/+; n = 10–14) and heterozygous FDNQ175 (FDNQ175/+; n = 15–19) female mice from 6 to 12 months of age tested at 2 month intervals. **(G)** Combined line graph summarizing changes in the mean ± SEM percent error (% error) in limb placement of wild-type (+/+) and heterozygous FDNQ175 (FDNQ175/+) male and female mice from 6 to 12 months of age tested at 2 month intervals. * indicates significant difference (p < 0.05) between age-matched and sex-matched wild-type (+/+) and heterozygous FDNQ175 (FDNQ175/+) mice. ^#^ indicates significant difference (p < 0.05) as compared with 6-month-old sex-matched wild-type (+/+) mice. ^Ψ^ indicates significant difference (p < 0.05) as compared with 6-month-old sex-matched heterozygous FDNQ175 (FDNQ175/+) mice

At 6 months of age, neither male nor female heterozygous FDNQ175 mice made significantly more errors in their limb placements than age- and sex-matched wild-type controls during horizontal ladder traversal (Fig. 3A). However, at 8 months of age, male heterozygous FDNQ175 mice developed deficits in their limb placements, as evidenced by their significantly higher number of mistakes on the horizontal ladder compared to male wild-type mice of the same age (Fig. 3B). Impairments in limb placements were also observed in 10- and 12-month-old male heterozygous FDNQ175 mice compared to age-matched male wild-type controls (Fig. 3C and D). Furthermore, the deficits in limb placement appeared to progress aggressively from the age of 6 months to 10 month and then plateaus (Fig. 3E). In comparison, the number of mistakes made by female heterozygous FDNQ175 mice during horizontal ladder traversal also showed an upward trend between the age of 6 months and 10 months (Fig. 3F and G). However, there is no statistically significant difference between the horizontal ladder performance of female heterozygous FDNQ175 mice and age-matched female wild-type mice across all four time points tested (Fig. 3A-D and F). In conclusion, HD-related deficits in limb coordination and placements appeared to be less pronounced in female heterozygous FDNQ175 mice than males based on the results of the horizontal ladder test.

### Heterozygous FDNQ175 show similar locomotor activity as wild-type mice

**Fig. 4:**
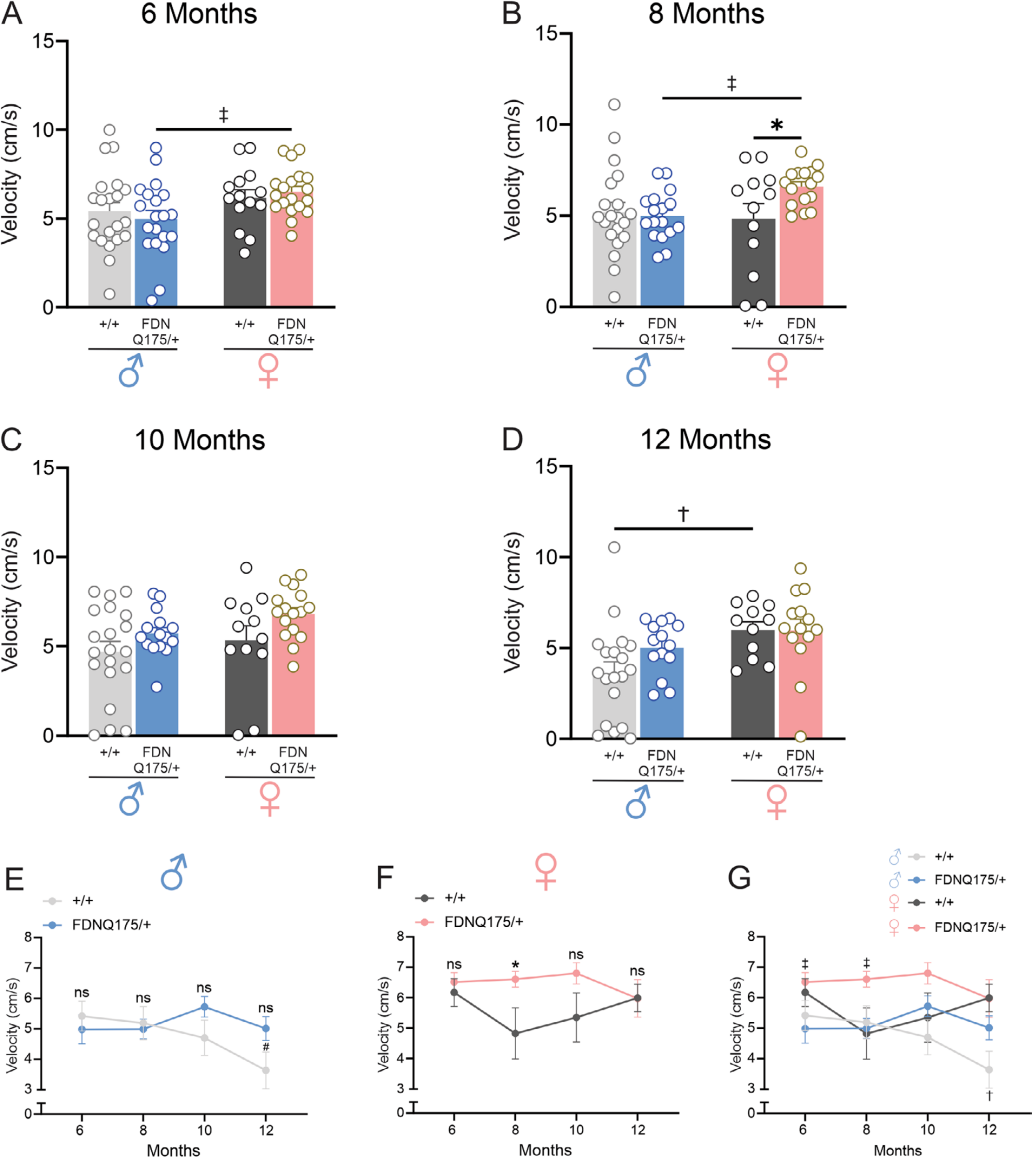
Locomotor activity of male and female wild-type and FDNQ175 mice from 6 to 12 months of age. Mean ± SEM average velocity (cm/s) of wild-type (+/+) and heterozygous FDNQ175 (FDNQ175/+) male and female mice at 6 months (n = 14–21 per group) **(A)**, 8 months (n = 12–20 per group) **(B)**, 10 months (n = 12–20 per group) **(C)**, and 12 months (n = 11–19 per group) **(D)** of age in an open-field arena. **(E)** Line graph summarizing changes in the mean ± SEM average velocity (cm/s) of wild-type (+/+; n = 19–21) and heterozygous FDNQ175 (FDNQ175/+; n = 14–20) male mice from 6 to 12 months of age tested at 2 month intervals. **(F)** Line graph summarizing changes in the mean ± SEM average velocity (cm/s) of wild-type (+/+; n = 11–14) and heterozygous FDNQ175 (FDNQ175/+; n = 14–19) female mice from 6 to 12 months of age tested at 2 month intervals. **(G)** Combined line graph summarizing changes in the mean ± SEM average velocity (cm/s) of wild-type (+/+) and heterozygous FDNQ175 (FDNQ175/+) male and female mice from 6 to 12 months of age tested at 2 month intervals. * indicates significant difference (p < 0.05) between age-matched and sex-matched wild-type (+/+) and heterozygous FDNQ175 (FDNQ175/+) mice. ^#^ indicates significant difference (p < 0.05) as compared with 6-month-old sex-matched wild-type (+/+) mice. † indicates significant difference (p < 0.05) between age-matched male and female wild-type (+/+) mice. ‡ indicates significant difference (p < 0.05) between age-matched male and female heterozygous FDNQ175 (FDNQ175/+) mice

**Fig. 5:**
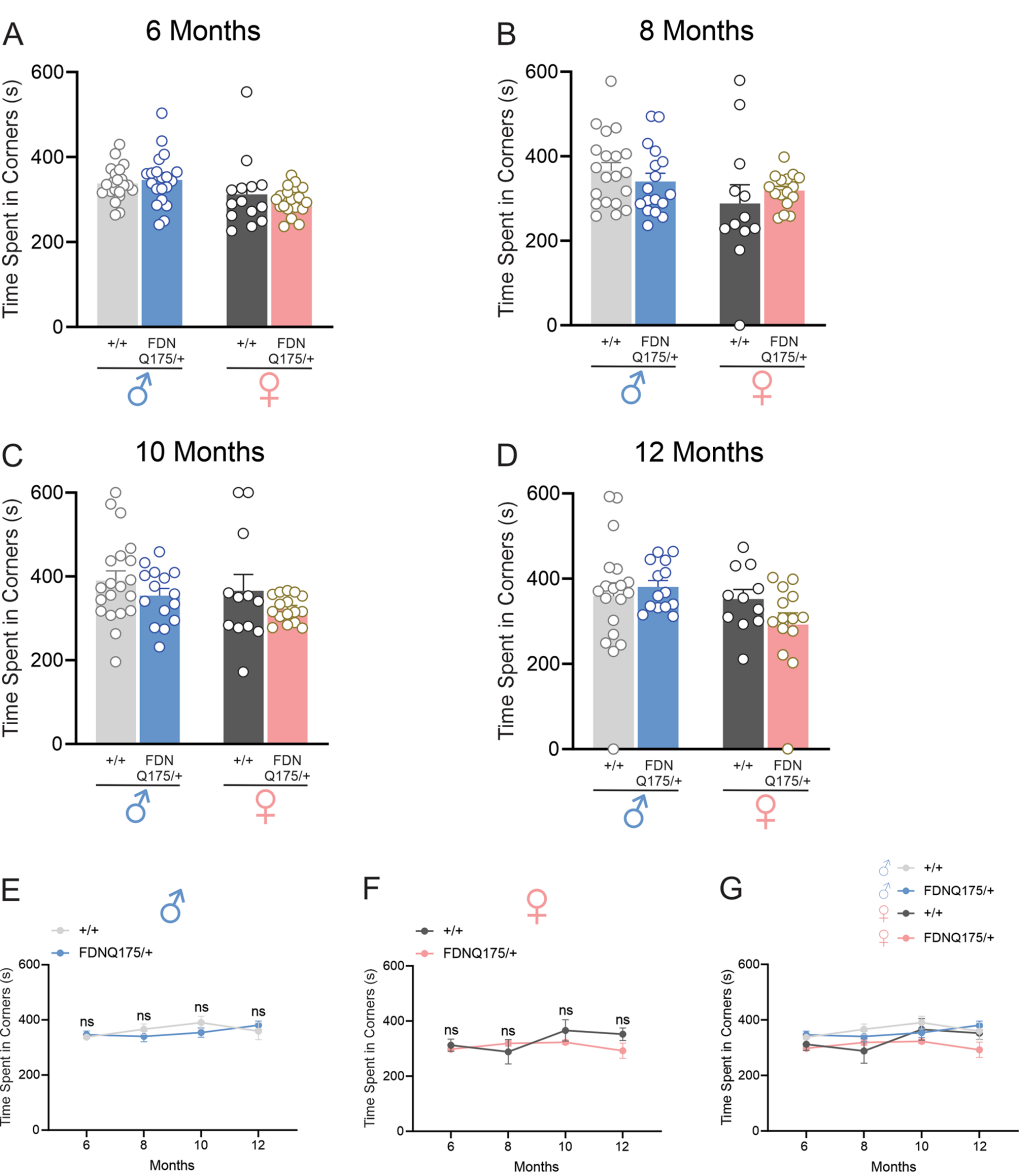
Time spent in four corners by male and female wild-type and FDNQ175 mice from 6 to 12 months of age. Mean ± SEM total time spent in four corners (s) of an open-field arena by wild-type (+/+) and heterozygous FDNQ175 (FDNQ175/+) male and female mice at 6 months (n = 14–21 per group) **(A)**, 8 months (n = 12–20 per group) **(B)**, 10 months (n = 12–20 per group) **(C)**, and 12 months (n = 11–19 per group) **(D)** of age. **(E)** Line graph summarizing changes in the mean ± SEM total time spent in four corners (s) of wild-type (+/+; n = 19–21) and heterozygous FDNQ175 (FDNQ175/+; n = 14–20) male mice from 6 to 12 months of age tested at 2 month intervals. **(F)** Line graph summarizing changes in the mean ± SEM total time spent in four corners (s) of wild-type (+/+; n = 11–14) and heterozygous FDNQ175 (FDNQ175/+; n = 14–19) female mice from 6 to 12 months of age tested at 2 month intervals. **(G)** Combined line graph summarizing changes in the mean ± SEM total time spent in four corners (s) of wild-type (+/+) and heterozygous FDNQ175 (FDNQ175/+) male and female mice from 6 to 12 months of age tested at 2 month intervals (ns: nonsignificant)

In addition to decline in motor functions, decrease in locomotor activity has been observed in several mouse models of HD, including the R6/2 and zQ175 mouse lines [13, 15–17]. Therefore, we examined the locomotor activity of heterozygous FDNQ175 mice at different ages. Locomotor activity in an open field arena, as measured by average velocity, remained similar between sex-matched wild-type and heterozygous FDNQ175 mice at 6 months of age (Fig. 4A). In contrast to their decline in grip strength and rotarod performance, heterozygous FDNQ175 mice did not show decreased locomotor activity as they aged. Indeed, with the exception of female mice at 8 months, both male and female heterozygous FDNQ175 mice showed similar levels of locomotor activity as age- and sex-matched wild-type controls across all time points tested in this study (Fig. 4A-G). Interestingly, female heterozygous FDNQ175 mice did show higher levels of locomotor activity than age-matched male heterozygous FDNQ175 mice at 6 months and 8 months of age (Fig. 4A, B and G). Moreover, male wild-type mice showed a trend of decreasing locomotor activity, and their average velocity at 12 months of age is significantly lower compared to their own 6-month data and 12-month-old female wild-type mice (Fig. 4E and G). We also assessed anxiety-like behaviour in FDNQ175 mice by calculating the time spent in four corners of the arena, as more anxious mice have a tendency to spend more time in the corners of the testing arena. Within the time frame we tested, both male and female heterozygous FDNQ175 mice spent similar amount of time as age- and sex-matched wild-type controls in the four corners on average (Fig. 5A-G). Taken together, heterozygous FDNQ175 mice of both sexes did not appear to develop discernible deficits in locomotor activity or anxiety-like behavior up to 12 months of age.

### Both female wild-type and heterozygous FDNQ175 mice develop deficits in cognitive function

**Fig. 6:**
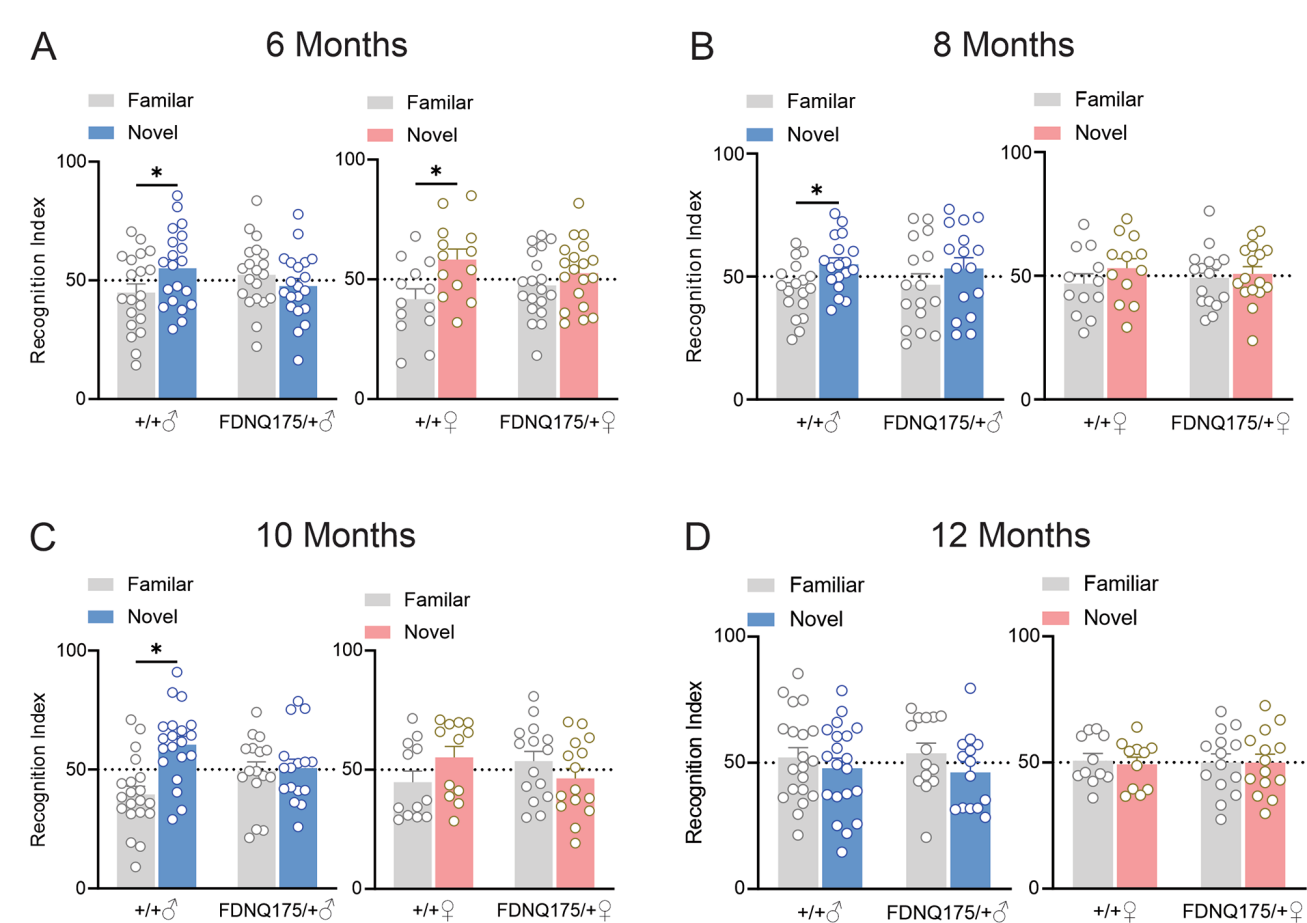
Novel object recognition in male and female wild-type and FDNQ175 mice from 6 to 12 months of age. Mean ± SEM of the recognition index, for exploring a novel object versus a familiar object on the second day of novel object recognition test, of wild-type (+/+) and heterozygous FDNQ175 (FDNQ175/+) male and female mice at 6 months (n = 13–20 per group) **(A)**, 8 months (n = 12–18 per group) **(B)**, 10 months (n = 12–20 per group) **(C)**, and 12 months (n = 11–20 per group) **(D)** of age. * indicates significant difference (p < 0.05) between recognition index of the familiar and the novel object

Cognitive impairments have been well documented in HD patients and can often appear prior to formal diagnosis by motor symptoms [18]. Similarly, cognitive deficits have been observed in mouse models of HD [13, 19]. In this study, we examined the development of cognitive decline in the heterozygous FDNQ175 mouse line. At 6 months of age, both male and female heterozygous FDNQ175 mice failed to distinguish between novel and familiar objects, whereas age-matched wild-type mice showed significantly higher preference to explore the novel object on day 2 of the novel object recognition test (Fig. 6A). Furthermore, both male and female heterozygous FDNQ175 continue to show inability to recognize the novel object in tests conducted at 8, 10 and 12 months of age (Fig. 6B-D). Unexpectedly, wild-type mice used in the study also exhibited decline in their cognitive functions. At 12 months of age, male wild-type mice no longer show preference towards the novel object (Fig. 6D). This surprising decline is observed even earlier in female wild-type mice as they explored both the novel and familiar objects equally starting at 8 months of age (Fig. 6B). The early loss of cognitive function in the novel object recognition test in wildtype mice limited our ability to examine the sex-specific progression of cognitive decline in FDNQ175 mice, especially with regard to females.

### FDNQ175 mice show progressive increase in huntingtin aggregate deposition

**Fig. 7:**
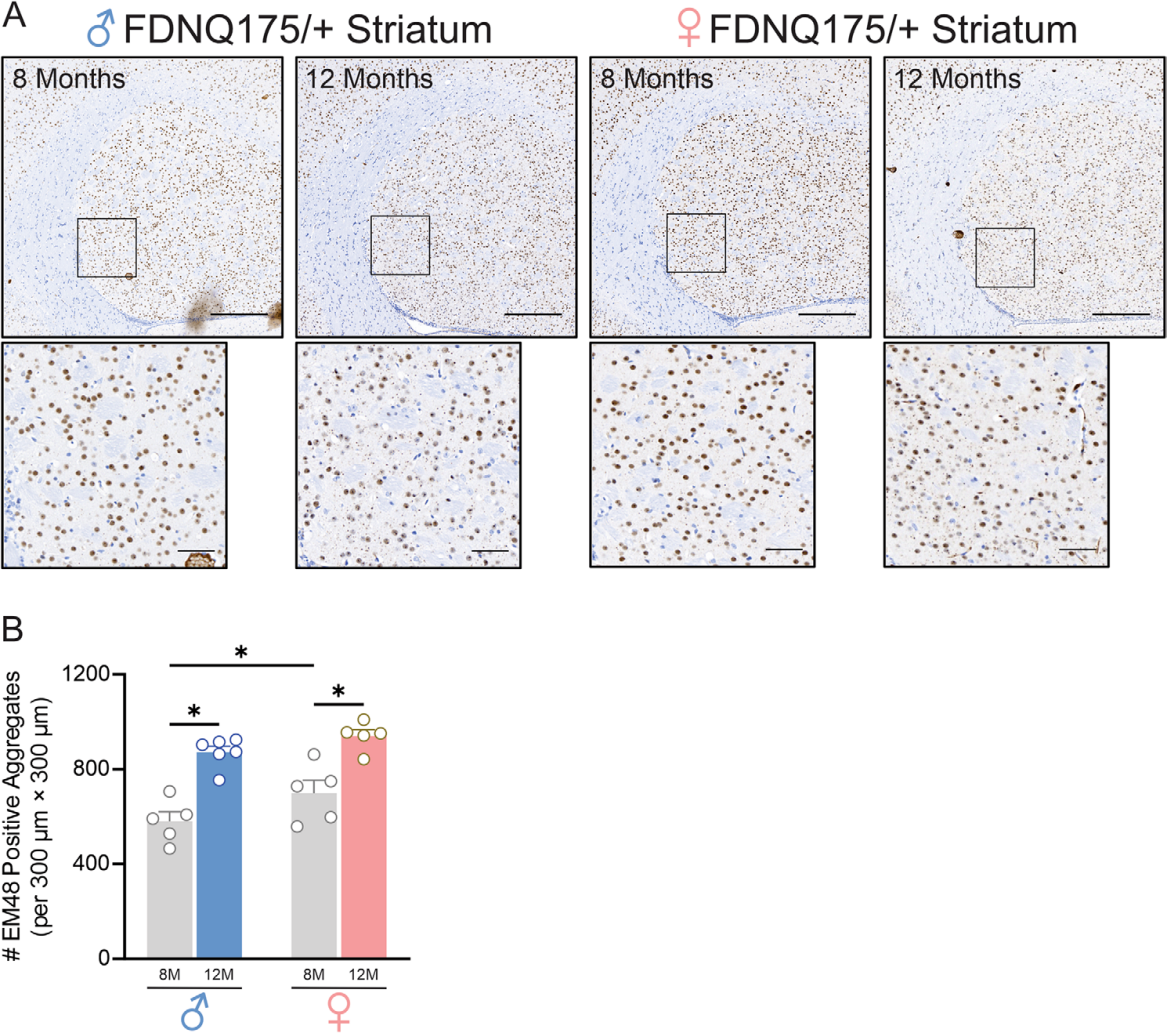
Deposition of mutant huntingtin aggregates increases with age in both male and female FDNQ175 mice. Representative images of staining for mutant huntingtin aggregates using the antibody EM48 **(A)** and quantification of the number of huntingtin aggregates **(B)** in striatal brain slices from 8- and 12-month-old male and female FDNQ175 mice. Scale bar, 300 μm for whole striatum and 50 μm for magnified areas. Data were quantified from two different 300 × 300 µm^2^ striatal regions of 6 sections per mouse and five independent mouse brains from each group were used for analysis. * indicates significant difference (p < 0.05)

The formation of cytoplasmic and intracellular aggregates is the key pathological hallmark of HD and these insoluble aggregates have been shown to disrupt normal cellular functions by trapping critical proteins into their matrix [4, 20]. We have previously detected insoluble mHtt aggregates in 15-month-old male and female heterozygous zQ175 mice [13, 15, 16]. Given the progressive nature of HD in humans, we examined whether the number of mutant huntingtin aggregates in the striatum of FDNQ175 mice increases as they age by staining for EM48 positive aggregates in 8- and 12-month-old mice. Both male and female FDNQ175 mice showed wide-spread mutant huntingtin aggregate deposition in the striatum at 8 months of age. The number of mutant huntingtin aggregates in 12-month-old male and female FDNQ175 mice is significantly higher than that of sex-matched 8-month-old FDNQ175 mice (Fig. 7A and B). Interestingly, at 8 months of age, female heterozygous FDNQ175 mice have significantly higher number of mutant huntingtin aggregates in the striatum than male heterozygous FDNQ175 mice (Fig. 7A and B). However, the level of insoluble aggregates was similar between male and female FDNQ175 mice at 12 months of age (Fig. 7A and B). Overall, we detected an age-dependent increase in the level of mutant huntingtin aggregates in FDNQ175 mice.

## Discussion

The creation of HD mouse models represents a critical step in the research and development of effective treatments for this devastating neurodegenerative disease. Among the different available mouse models, the R6/2 line is the most commonly used for preclinical drug research due to its rapid and aggressive phenotypic progression. However, the R6/2 mouse only expresses exon 1 of the human mutant huntingtin gene and its early disease onset makes it more suitable for modelling juvenile HD. On the other hand, the zQ175 knock-in mouse model of HD represents a more genetically accurate replication of the human condition since its mutant huntingtin expression is under the control of the endogenous promoter and it shows robust phenotype on a heterozygous background [8]. The novel FDNQ175 mouse line attempts to improve on the zQ175 model by removing the neo cassette to increase mutant huntingtin expression and using the FVB/N background strain to increase susceptibility to neurodegeneration [9]. In this study, we performed longitudinal assessment of the behavioral deficits and neuropathology in heterozygous FDNQ175 mice of both sexes. We found that both male and female FDNQ175 mice showed lower grip strength and cognitive deficits starting 6 months of age. However, we did not detect hypoactivity and elevated anxiety in FDNQ175 mice. More interestingly, female FDNQ175 mice appeared less susceptible to decline in motor function than male mice but showed higher levels of insoluble mHtt aggregates at a younger age.

Growing evidence suggest that sex is a potential contributing factor in the pathophysiology of neurodegenerative diseases [13, 15, 16]. Here, we tested both motor and disease pathology progression in FDNQ175 mice to determine whether sex influences the onset and development of HD symptoms in this mouse model. We observed that deficits in limb placements and coordination, as measured by the horizontal ladder test, but not grip strength, developed in a sex-dependent manner. This is consistent with the trend observed in Hdh350/+ mice, where gait abnormalities and reduction in motor coordination are detected in only males and not females, but the loss of grip strength is not sex-dependent [21]. Interestingly, female heterozygous FDNQ175 mice have higher levels of insoluble mHtt aggregates in the striatum than male heterozygous FDNQ175 mice at 8 months of age, when only male but not female heterozygous FDNQ175 mice showed significantly worse horizontal ladder test performance. Some studies have suggested that insoluble aggregates can play a protective role by sequestering the more toxic protein species, most notably the N-terminal fragments of mutant huntingtin, and thus reducing their toxicity [22]. At 12 months of age, the levels of mHtt aggregates reached a similar level in male and female striatum. It would be interesting in the future to examine whether female heterozygous FDNQ175 mice begin to perform worse than female wild-type mice in the horizontal ladder test after 12 months of age. This could help determine whether insoluble mHtt aggregates play a neuroprotective or neurotoxic role in HD, which remains debatable.

The lack of sex-specific differences in the decline of grip strength may be contributed to the fact that grip strength is found to be controlled, to a large extent, by globus pallidus internus (GPi) and the subthalamic nucleus (STN) [23]. It is possible that HD pathology in these two basal ganglia nuclei is different from that of the striatum, which could explain why the onset of grip strength deficits are the same between male and female mice despite the sex-dependent changes observed in their striatum.

Anxiety and cognitive decline are key symptoms of HD and can even appear years before disease onset [24]. Here, we did not detect anxiety-like behaviour or reduced locomotor activity in FDNQ175 mice up to 12 months of age, although female FDNQ175 mice had higher levels of locomotor activity than male FDNQ175 mice at younger ages. In a previous study conducted with these mice, decreased open field locomotor activity was not detected in heterozygous FDNQ175 mice up to 9 months of age [9]. Here, we showed that even 12-month-old heterozygous FDNQ175 mice do not have reduced locomotor activity. On the other hand, the onset of cognitive deficits in both male and female FDNQ175 mice is consistent with the previous report in which 6-month-old heterozygous FDNQ175 mice did not show a preference for the novel object [9]. However, our male and female wild-type mice showed an unexpected early decline in cognitive function at 12 months and 8 months of age, respectively. The surprising decline in the cognitive function of wild-type mice may be caused by inherent vulnerabilities of the FVB/N strain. It was previously reported that FVB/N mice are at risk of sudden death and nervous system lesion due to a pathological syndrome called “space cadet” [25, 26]. The brain lesions are characterized by neurodegenerations in the thalamus, cerebral cortex and hippocampus [25]. More importantly, female FVB/N mice are shown to be more predisposed to this pathological condition than males [25]. Therefore, it is possible that the early decline in the cognitive function of our female wild-type controls is caused by the aforementioned syndrome, as the hippocampus is a brain region well-known to be important for learning and memory [27].

Overall, it appears that sex does have a significant influence on the decline of motor coordination and limb function associated with HD. One area worth exploring in the future is the action of sex hormones in HD. Notably, membrane estrogen receptors are coupled to metabotropic glutamate receptor 5 (mGluR5) in female rat striatum and can activate mGluR5 signalling in the presence of estradiol [28]. We have previously demonstrated that mGluR5 interacts with mHtt and its downstream signalling is implicated in HD pathogenesis through complex mechanisms that are still not fully understood [13, 29, 30]. Furthermore, estrogen has been found to alter the probability of glutamate release, the activity of NMDA receptors and the excitability of medium spiny neurons, all of which are critical factors of excitotoxicity in HD [31–33]. In fact, it was discovered that estrogen regulates the probability of glutamate release through distinct mechanisms in male and female rats [34]. Therefore, glutamatergic signalling in HD brains could be heavily influenced by the activity of sex hormone receptors, leading to sex-specific differences in HD symptoms and progression.

One clear obstacle in determining the influence of sex in HD pathogenesis and progression is the somewhat contradictory data obtained from HD mouse models versus HD patients. Among HD patients, women tend to suffer from more severe motor and cognitive deficits [35–37]. In contrast, studies done using HD mouse models suggest that female HD mice are actually more resilient [10, 21]. For instance, circadian disruption and behaviour fragmentation were found to be less severe in female BACHD mice than males [38]. Furthermore, male mouse striatum has a lower level of extracellular ascorbate, which correlated with a more severe phenotype, and a higher level of disruption in neuroprotective nitric oxide synthase activity than female [11, 39, 40]. The discrepancies we see between human and mouse could be caused by a variety of factors, including subtle differences between human and mouse huntingtin gene, dissimilarities in the gene’s promoter and the extremely long polyglutamine tracts in some models that far exceed the average length reported in humans [7]. It would be interesting in the future to explore these plausible causes and their effects on neuroprotective or neurotoxic mechanisms in HD patients, which could explain the sex-specific differences seen in humans. Other non-human primate models of HD have been developed, such as the rhesus macaque model that expresses polygutamaine-expanded mHtt [41]. Compared to mice, it is possible that higher primates will better replicate the disease physiology and pathophysiology observed in humans and will be more suitable for studying sex-specific differences in HD [41].

In conclusion, we have shown that heterozygous FDNQ175 mice develop robust HD phenotypes in various behavioural tests at 6 months of age. Furthermore, consistent with data from other HD mouse models, female FDNQ175 mice appeared to be less vulnerable to HD-related impairments in limb functions than male mice. The mechanism(s) underlying the differences between male and female FDNQ175 mice remains unclear. We have previously demonstrated that pharmacological agents targeting post-synaptic mGluR5 signalling show different efficacies in male versus female mouse models of neurodegenerative diseases, suggesting that glutamate signalling plays a role on sex-specific differences and should be explored in the future [15, 16, 42]. Here, we provide further evidence that sex is a major contributing factor in the pathophysiology of HD and emphasize the importance of taking it into account when designing novel approaches for treatment.

## Data Availability

All data generated or analyzed during this study are included in this published article.
